# Case Report: High-Level MET Amplification as a Resistance Mechanism of ROS1-Tyrosine Kinase Inhibitors in ROS1-Rearranged Non-Small Cell Lung Cancer

**DOI:** 10.3389/fonc.2021.645224

**Published:** 2021-05-13

**Authors:** Jiangping Yang, Ping Zhou, Min Yu, Yan Zhang

**Affiliations:** ^1^ Department of Thoracic Oncology, Cancer Center, State Key Laboratory of Biotherapy, West China Hospital, West China Medical School, Sichuan University, Chengdu, China; ^2^ Department of Pathology, West China Hospital, Sichuan University, Chengdu, China

**Keywords:** MET amplification, ROS1, crizotinib, resistance, non-small cell lung cancer

## Abstract

**Background:**

Although C-ros oncogene 1 (ROS1) targeted therapies have demonstrated remarkable efficacy in ROS1-rearranged non-small cell lung cancer (NSCLC), patients inevitably develop resistance to ROS1-tyrosine kinase inhibitors (TKIs). Commonly acquired resistance mechanisms include a second mutation of the ROS1 kinase domain and activation of bypass signaling pathways. However, MMNG HOS Transforming gene (MET) amplification has not been reported as a novel mechanism of ROS1-TKIs resistance.

**Case Presentation:**

We report a case of a 62-year-old man diagnosed with ROS1-rearranged metastatic lung adenocarcinoma, who received first-line treatment with crizotinib for 19 months. During the course of disease, the primary lung tumor was under control while the brain metastasis progressed despite the treatment with lorlatinib. The biopsy and genetic tests of the metastatic brain tumor showed a high level of MET amplification (32 copies). However, fluorescence *in situ* hybridization of the primary cancer showed no MET amplification, suggesting that MET amplification may be associated with an acquired resistance to ROS1-TKIs.

**Summary:**

This case suggested that MET amplification could be explored as a potential mechanism for developing ROS1-TKIs resistance. Combination treatment with highly potent and selective MET-TKIs warrants further investigations.

## Introduction

C-ros oncogene 1 (ROS1) has been identified as an oncogenic driver in 1–2% of non-small cell lung cancer (NSCLC) (1). Patients with ROS1-rearranged lung cancer significantly benefit from treatment with ROS1 tyrosine kinase inhibitors (TKIs) (2), such as crizotinib, with an overall response rate (ORR) of 72% and a progression free survival (PFS) of approximately 19.3 months (3). However, following initial positive responses to crizotinib, a large number of patients with ROS1-rearranged NSCLC experience progression of disease due to the occurrence of resistance. The most common mechanism of acquired resistance to TKIs is secondary mutation of the ROS1 kinase domain, such as G2032R, L2155S, and S1986F/Y, which decreases the potency of kinase inhibition. Furthermore, the activation of bypass signaling pathways, including EGFR, KIT, and MAPK, have been identified as another mechanism of resistance in ROS1-rearranged cancers and accounts for about 45% of the crizotinib-resistance ROS1-rearranged NSCLC (4, 5). The treatment options, including switching drugs and combination therapies, can vary based on the different underlying mechanisms.

In this study, we reported a case wherein a high level of MMNG HOS Transforming gene (MET) amplification was found at the time of progression, as a potential mechanism for ROS1-TKIs resistance in NSCLC.

## Case Description

**Figure 1:**
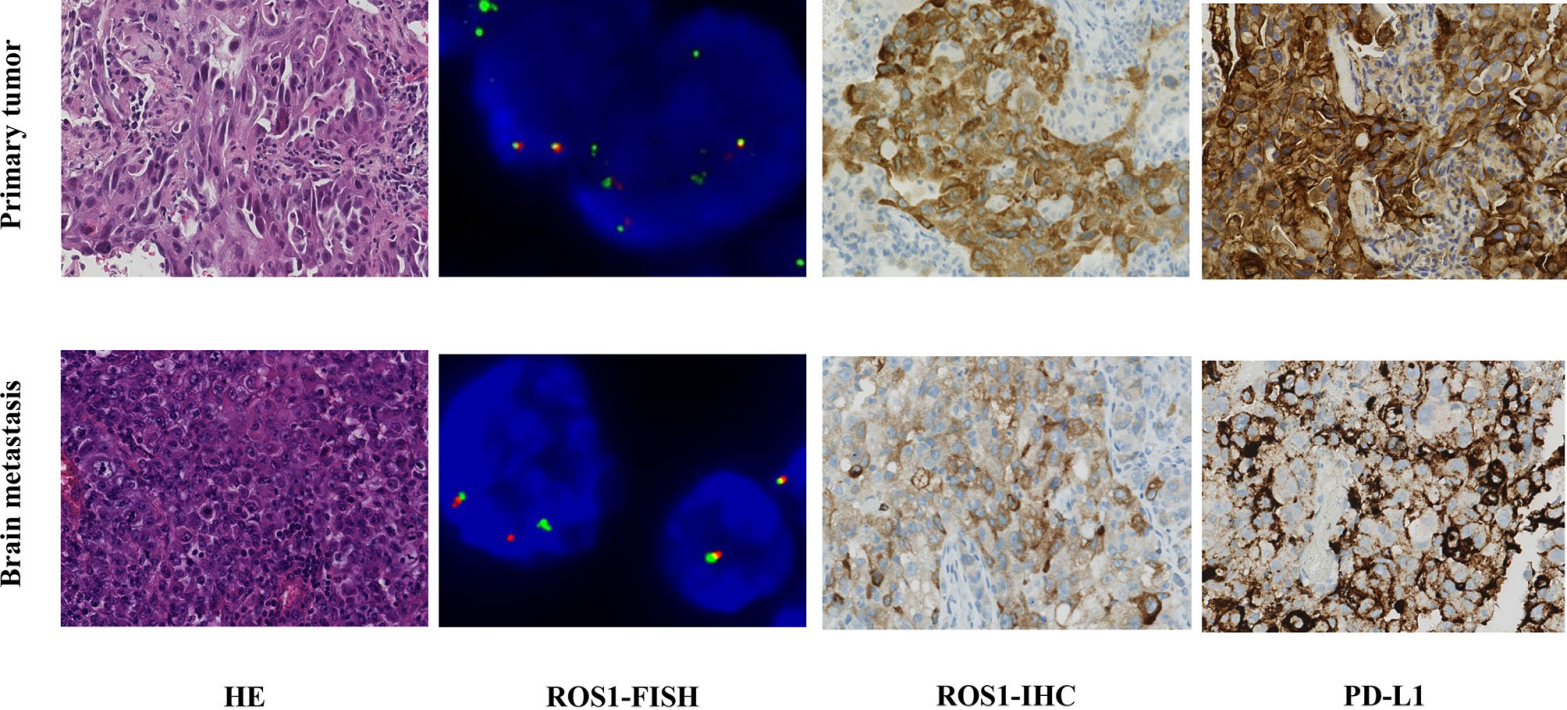
Comparison of findings from biopsy of the primary lung tumor and metastatic brain tumor. Hematoxylin and eosin staining (HE ×40) of the primary lung adenocarcinoma was positive for ROS1 and PD-L1 (> 90%) by immunohistochemical (IHC ×40). The metastatic brain tumor showed poorly differentiated carcinoma by HE, which was partly positive for ROS1 and PD-L1 (40%) by IHC. ROS1 rearrangement was confirmed by FISH (>15% of the cells demonstrate split or single 3′ signals).

A 62-year-old male nonsmoker presented at our hospital in January 2018. He complained of a one-month history of right chest and back discomfort with no noted triggers. Imaging examinations showed a 2.3 cm mass in his right middle lobe, with metastasis in the right hilar and mediastinal lymph nodes as well as left ilium. A solitary brain metastasis in the left frontal lobe was also noted in magnetic resonance imaging (MRI). Pulmonary biopsy revealed lung adenocarcinoma stage IV (T1cN2M1c). Immunohistochemistry (IHC) results of the tumor tissue were positive for ROS1 and PD-L1 (> 90%) but negative for anaplastic lymphoma kinase (ALK) (**Figure 1**). ROS1 rearrangement was then confirmed by fluorescence *in situ* hybridization (FISH) (**Figure 1**).

**Figure 2:**
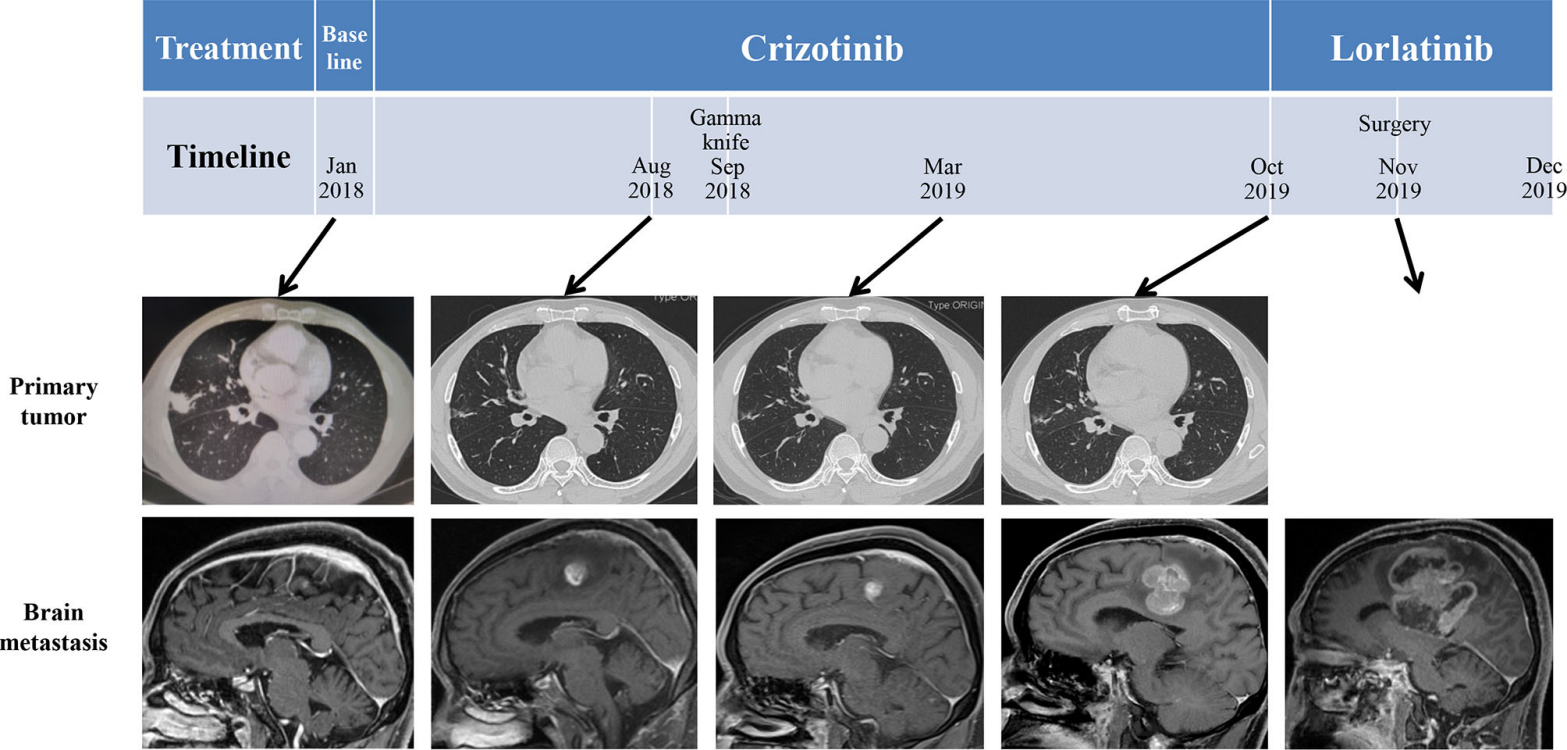
Schematic diagram of the course of the patient’s disease. Imaging before treatment showed a lung mass in the right middle lobe and left frontal lobe metastasis. After administration of crizotinib, the primary tumor size shrank; however, left frontal lobe metastasis was larger.

**Figure 3:**
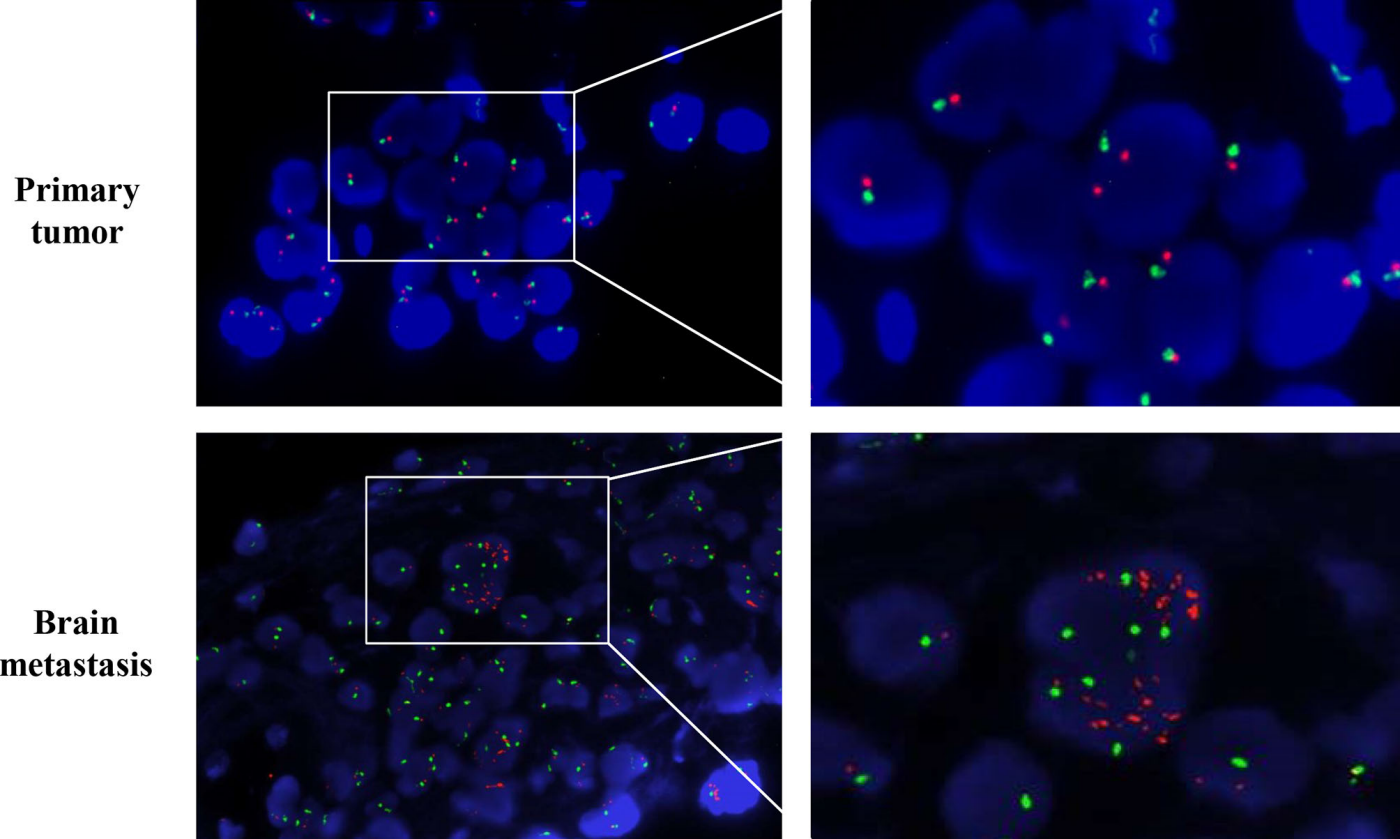
MMNG HOS transforming gene (MET) amplification test by fluorescence *in situ* hybridization (FISH). MET is represented by a single red dot, and centromere probe of chromosome 7 (CEP7) is represented by a single green dot. Amplification test by FISH revealed no MET amplification (MET/CEP7 ratio <2.0 and MET per cell<5) in the primary lung tumor per-crizotinib but a cluster MET amplification (MET/CEP7 ratio ≥2.0 and MET per cell≥5) in the metastatic brain tumor post-crizotinib.

The patient was given first-line treatment with crizotinib 250mg twice daily from February 2018. This achieved a partial response with good tolerance. Six months later, he felt dizzy, right lower extremity weakness, and unsteadiness. Imaging showed a controlled primary lung lesion but a progressing brain lesion. Therefore, the patient continued to receive crizotinib and underwent concurrent gamma knife radiosurgery for the intracranial lesion. Six cycles of combination therapy with crizotinib and bevacizumab were initiated in March 2019. Approximately 19 months after crizotinib treatment was initiated, he reported dizziness and progressive weakness of his right lower limb. MRI confirmed disease progression and significant edema in the brain in October. A computed tomography (CT) scan of the chest showed the primary lesion still under control. The patient then received lorlatinib 75mg once daily, but his symptoms were not relieved. Instead, his dizziness and the muscular weakness in both lower extremities worsened. New symptoms, such as headache, convulsions, sluggishness, and unresponsiveness developed. A repeated brain-MRI showed rapid brain tumor progression, severe brain edema, and cerebral midline deviation (**Figure 2**). Then, an emergency brain metastasis surgery was performed, and the pathological tests demonstrated the presence of adenocarcinoma from lung cancer. The specimen was further analyzed *via* next-generation sequencing (NGS; FoundationOne CDx). Unfortunately, the patient succumbed to the intracranial hypertension and cerebral hernia before the NGS report, which showed a high level of MET amplification (32 copies). FISH demonstrated a cluster of MET signals congruent with the NGS result (**Figure 3**). To investigate whether MET amplification was acquired after TKI treatment, the primary lung lesion was reanalyzed by FISH, and the result was negative for MET amplification (**Figure 3**).

## Discussion

MET amplification activates a bypass signaling pathway that is responsible for resistance to EGFR and ALK TKIs (6, 7). However, its role in ROS1-TKIs has never been reported. To our knowledge, this is the first report suggesting MET amplification can serve as a mechanism for acquired resistance to ROS1-TKIs. In the present case, the results of pathological analysis confirmed the acquisition of MET amplification after crizotinib therapy using NGS and FISH. The immediate post-crizotinib brain tumor specimen showed a high level of MET amplification (32 copies) in contrast to the MET-negative pre-crizotinib primary lung tumor. Combining the patient’s initial response to crizotinib and subsequent disease progression facts, speculation arises that MET amplification contributed to acquired ROS1-TKI resistance. In addition, the rapid growth of the tumor within a month post-lorlatinib indicated that high-level MET amplification is a potentially potent oncogenic driver. However, the patient was not able to receive treatment with selective MET inhibitors to validate and overcome this resistance mechanism.

Crizotinib, a multitargeted ALK/ROS1/MET inhibitor, was the first TKI approved by the FDA for the treatment of ROS1-rearranged advanced NSCLC in 2016 (8). It inhibits ATP-dependent cellular functions by binding to the respective protein kinase domains, leading to potent ALK, ROS1, and MET suppression and generating positive clinical effects (9). The observations of several cases indicated that NSCLC patients with MET amplification could benefit from crizotinib (10–12). A previous report described two patients with high-level MET amplification NSCLC (MET/CEP7 ratio≥5) responding to crizotinib in the absence of detectable exon 14 alterations (10). A recent case reported that MET D1228N mutation might be a mechanism of resistance to crizotinib in ROS1 fusion NSCLC (13). The resistances to ROS1-TKIs of both cases may have been due to variations of MET, but *via* different mechanisms: MET secondary mutation and MET amplification. Further studies are needed to confirm these finding.

Recently, the activity of crizotinib against MET-amplified NSCLC has been demonstrated, but which was at a lower level than that seen against ROS1 (14–16). In the phase II AcSé trail, which evaluated the efficacy of crizotinib in MET amplification and ROS1-positive NSCLC, the best ORR during treatment was 32% in the MET amplification cohort and 69.4% in the ROS1-positive cohort (15). Another phase II study reported that crizotinib produced an ORR of 27% with a median PFS of 4.4 months in MET amplification patients, while the ORR and median PFS were 65% and 22.8 months, respectively, in ROS1-rearranged patients (16). Likewise, the results of our current case confirmed the unfavorable prognosis of MET-amplification NSCLC with crizotinib therapy. The lower activity of crizotinib in overcoming MET amplification may be due to its higher IC_50_ for MET amplification (0.58 µM) compared to that for ROS1 (31nM for SDC4–ROS1 fusion) (17, 18). The IC_50_ of crizotinib for MET *in vitro* was approximately 4nM (19), which was much lower to that for MET amplification. It could be another reason for the decreased sensitivity of crizotinib in patients with MET amplification. In addition, it was reported that the CSF-to-plasma ratio of crizotinib concentration was 0.0026 (20), which implied a poor blood-brain barrier penetration. These factors may explain why acquired MET amplification was found in the brain lesion but not observed in extracranial sites with the treatment of crizotinib.

In previous studies, patients harboring acquired MET amplifications after disease progression benefited from combination therapy that targeted both the primary driver and MET gene (21). A phase Ib/II study found that the efficacy of combination therapy (capmatinib and gefitinib) increased along with the MET copy number for patients with EGFR-mutated, MET-amplified NSCLC (22). This suggested that the likelihood of benefiting from MET-TKIs rises as the level of MET amplification increases. Therefore, combination therapy with more potent selective MET-TKIs should be considered. In addition, the primary lung tumor and brain metastasis tumor were both positive for PD-L1 (greater than 90% and 40%, respectively), suggesting that the patient may benefit from a combination of immunotherapy and target therapy. Furthermore, the variation of the PD-L1 and MET during the disease progression revealed the highly heterogeneous character of the patient’s cancer. Therefore, repeated genetic testing during the treatment is crucial.

In summary, this case suggested that MET amplification may be an important mechanism for acquired resistance to ROS1-TKIs. The combination of crizotinib and a more potent MET inhibitor should be investigated post-crizotinib progression in ROS1-rearranged NSCLC patients who harbored acquired MET amplification.

## Data Availability Statement

The original contributions presented in the study are included in the article/supplementary material. Further inquiries can be directed to the corresponding author.

## Author Contributions

JY: collected the clinical data and wrote/edited the final manuscript. PZ: analyzed and interpreted the histological examination, immunohistochemistry stains, and fluorescence *in situ* hybridization. MY: patient care and case presentation. YZ: conceptualization, writing-review and editing. All authors contributed to the article and approved the submitted version.

## Funding

The authors acknowledge financial supports from the 1·3·5 project for disciplines of excellence–Clinical Research Incubation Project, West China Hospital, Sichuan University (Grant No. 2019HXFH062), and from the National Natural Science Foundation of China (Grant No. 81402561).

## Conflict of Interest

The authors declare that the research was conducted in the absence of any commercial or financial relationships that could be construed as a potential conflict of interest.

## References

[1] Bergethon K Shaw AT Ou SH Katayama R Lovly CM McDonald NT. ROS1 Rearrangements Define a Unique Molecular Class of Lung Cancers. J Clin Oncol (2012) 30:863–70. 10.1200/JCO.2011.35.6345 PMC3295572 22215748

[2] Shaw AT Ou SH Bang YJ Camidge DR Solomon BJ Salgia R. Crizotinib in ROS1-rearranged non-Small-Cell Lung Cancer. N Engl J Med (2014) 371:1963–71. 10.1056/NEJMoa1406766 PMC4264527 25264305

[3] Shaw AT Riely GJ Bang YJ Kim DW Camidge DR Solomon BJ. Crizotinib in ROS1-rearranged Advanced non-Small-Cell Lung Cancer (NSCLC): Updated Results, Including Overall Survival, From PROFILE 1001. Ann Oncol (2019) 30:1121–6. 10.1093/annonc/mdz131 PMC6637370 30980071

[4] Drilon A Jenkins C Iyer S Schoenfeld A Keddy C Davare MA. ROS1-Dependent Cancers - Biology, Diagnostics and Therapeutics. Nat Rev Clin Oncol (2021) 18:35–55. 10.1038/s41571-020-0408-9 32760015 PMC8830365

[5] Dagogo-Jack I Rooney M Nagy RJ Lin JJ Chin E Ferris LA. Molecular Analysis of Plasma From Patients With ROS1-Positive Nsclc. J Thorac Oncol (2019) 14:816–24. 10.1016/j.jtho.2019.01.009 PMC6486857 30664990

[6] Engelman JA Zejnullahu K Mitsudomi T Song Y Hyland C Park JO. MET Amplification Leads to Gefitinib Resistance in Lung Cancer by Activating ERBB3 Signaling. Science (2007) 316:1039–43. 10.1126/science.1141478 17463250

[7] Guo R Luo J Chang J Rekhtman N Arcila M Drilon A. MET-Dependent Solid Tumours - Molecular Diagnosis and Targeted Therapy. Nat Rev Clin Oncol (2020) 17:569–87. 10.1038/s41571-020-0377-z PMC7478851 32514147

[8] FDA Expands Use of Xalkori to Treat Rare Form of Advanced non-Small Cell Lung Cancer (2016). Available at: https://www.fda.gov/news-events/press-announcements/fda-expands-use-xalkori-treat-rare-form-advanced-non-small-cell-lung-cancer (Accessed 04 Aug 2020).

[9] Roskoski R Jr. ROS1 Protein-Tyrosine Kinase Inhibitors in the Treatment of ROS1 Fusion Protein-Driven non-Small Cell Lung Cancers. Pharmacol Res (2017) 121:202–12. 10.1016/j.phrs.2017.04.022 28465216

[10] Caparica R Yen CT Coudry R Ou SI Varella-Garcia M Camidge DR. Responses to Crizotinib can Occur in High-Level Met-Amplified Non-Small Cell Lung Cancer Independent of MET Exon 14 Alterations. J Thorac Oncol (2017) 12:141–4. 10.1016/j.jtho.2016.09.116 27664533

[11] Deng L Kiedrowski LA Ravera E Cheng H Halmos B. Response to Dual Crizotinib and Osimertinib Treatment in a Lung Cancer Patient With MET Amplification Detected by Liquid Biopsy Who Acquired Secondary Resistance to EGFR Tyrosine Kinase Inhibition. J Thorac Oncol (2018) 13:e169–72. 10.1016/j.jtho.2018.04.007 30166014

[12] Ou SHI Kwak EL Siwak-Tapp C Dy J Bergethon K Clark JW. Activity of Crizotinib (PF02341066), a Dual Mesenchymal-Epithelial Transition (MET) and Anaplastic Lymphoma Kinase (Alk) Inhibitor, in a Non-small Cell Lung Cancer Patient With De Novo MET Amplification. J Thoracic Oncol (2011) 6:942–6. 10.1097/JTO.0b013e31821528d3 21623265

[13] Wang Y Chen Z Han X Li J Guo H Shi J. Acquired MET D1228n Mutations Mediate Crizotinib Resistance in Lung Adenocarcinoma With ROS1 Fusion: A Case Report. Oncologist (2021) 26:178–81. 10.1002/onco.13545 PMC7930416 33000474

[14] Camidge DR Otterson GA Clark JW Ignatius Ou S-H Weiss J Ades S. Crizotinib in Patients With MET-Amplified NSCLC. J Thoracic Oncol (2021) S1556-0864(21)01710-X. 10.1016/j.jtho.2021.02.010 33676017

[15] Moro-Sibilot D Cozic N Perol M Mazieres J Otto J Souquet PJ. Crizotinib in c-MET- or ROS1-positive NSCLC: Results of the AcSe Phase II Trial. Ann Oncol (2019) 30:1985–91. 10.1093/annonc/mdz407 31584608

[16] Landi L Chiari R Tiseo M D’Inca F Dazzi C Chella A. Crizotinib in MET-Deregulated or ROS1-Rearranged Pretreated Non-Small Cell Lung Cancer (Metros): A Phase II, Prospective, Multicenter, Two-Arms Trial. Clin Cancer Res (2019) 25:7312–9. 10.1158/1078-0432.CCR-19-0994 31416808

[17] Chiba M Togashi Y Tomida S Mizuuchi H Nakamura Y Banno E. MEK Inhibitors Against MET-amplified non-Small Cell Lung Cancer. Int J Oncol (2016) 49:2236–44. 10.3892/ijo.2016.3736 PMC5118002 27748834

[18] Davies KD Le AT Theodoro MF Skokan MC Aisner DL Berge EM. Identifying and Targeting ROS1 Gene Fusions in non-Small Cell Lung Cancer. Clin Cancer Res (2012) 18:4570–9. 10.1158/1078-0432.CCR-12-0550 PMC3703205 22919003

[19] Vansteenkiste JF Van De Kerkhove C Wauters E Van Mol P. Capmatinib for the Treatment of non-Small Cell Lung Cancer. Expert Rev Anticancer Ther (2019) 19:659–71. 10.1080/14737140.2019.1643239 31368815

[20] Costa DB Kobayashi S Pandya SS Yeo WL Shen Z Tan W. CSF Concentration of the Anaplastic Lymphoma Kinase Inhibitor Crizotinib. J Clin Oncol (2011) 29:e443–445. 10.1200/JCO.2010.34.1313 21422405

[21] Sequist LV Han JY Ahn MJ Cho BC Yu H Kim SW. Osimertinib Plus Savolitinib in Patients With EGFR Mutation-Positive, MET-amplified, non-Small-Cell Lung Cancer After Progression on EGFR Tyrosine Kinase Inhibitors: Interim Results From a Multicentre, Open-Label, Phase 1b Study. Lancet Oncol (2020) 21:373–86. 10.1016/S1470-2045(19)30785-5 32027846

[22] Wu YL Zhang L Kim DW Liu X Lee DH Yang JC. Phase Ib/II Study of Capmatinib (Inc280) Plus Gefitinib After Failure of Epidermal Growth Factor Receptor (Egfr) Inhibitor Therapy in Patients With EGFR-Mutated, Met Factor-Dysregulated non-Small-Cell Lung Cancer. J Clin Oncol (2018) 36:3101–9. 10.1200/JCO.2018.77.7326 30156984

